# Large Area Monolayer Graphene Transfer in Ultra-High Vacuum

**DOI:** 10.1021/acs.jpcc.4c08196

**Published:** 2025-04-10

**Authors:** Darius Merk, Stefano Rusponi, Harald Brune

**Affiliations:** Institute of Physics, École Polytechnique Fédérale de Lausanne (EPFL), Station 3, Lausanne CH-1015, Switzerland

## Abstract

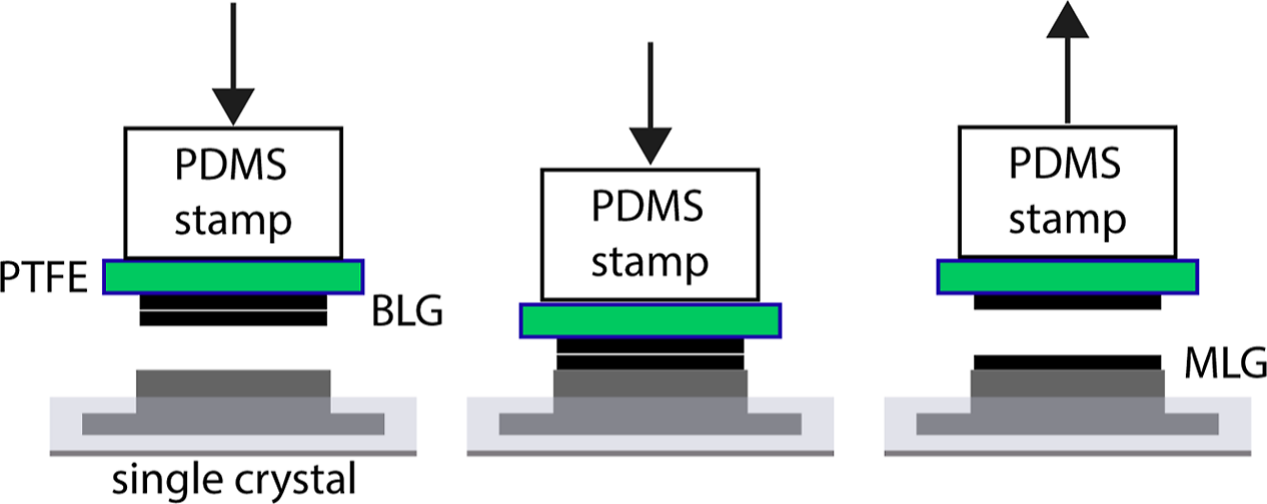

Graphene transfer methods either employ support layers that have to be removed after transfer, giving rise to impurities, or are based on delamination from bulk crystals, yielding only small flakes with various thicknesses. We present a graphene transfer method overcoming these disadvantages and working under ultrahigh vacuum. It is based on wafer bonding and uses a Teflon-supported graphene bilayer as a source. We demonstrate transfer of one graphene monolayer onto atomically clean Ir(111) and Cu(100) single-crystal surfaces over 5 × 5 mm^2^ large areas. Auger electron spectroscopy reveals that 70–100% of a graphene monolayer is transferred and that this layer is free from chemical defects, even within the detection limit of synchrotron-based X-ray absorption spectroscopy. Raman and X-ray absorption spectroscopy evidence high structural quality of the transferred graphene, and scanning tunneling microscopy shows the same moiré structure of graphene on Ir(111) that is obtained for chemical vapor deposition on that substrate. We show the versatility of our approach by creating a graphene bilayer on Ir(111). Our method enables us to cap entire surfaces in ultrahigh vacuum with a monolayer of clean 2D material, either for sealing them from the environment or for the creation of novel 3D metamaterials by sequential epitaxial growth and graphene transfer.

## Introduction

The growth of graphene on single-crystal substrates has been brought to maturity.^1,2^ Chemical vapor deposition (CVD) is the most common method and yields cm-sized monodomain single-crystal graphene on Ir(110)^3^ and wafer-scale graphene on hydrogen-terminated Ge(110)^4^ and on single-crystal metal films grown on sapphire wafers.^5−8^ However, generally, graphene does not show its targeted properties when chemically bound to its growth substrate, requiring its transfer to another target surface. Also, building graphene van der Waals (vdW) homo-^9−17^ and heterostructures,^18−21^ as well as graphene-based electronic devices,^22−25^ requires graphene transfer.

Commonly, this is achieved using support layers preventing graphene from breaking or rolling up during the transfer process.^26,27^ Typically, Poly(methyl methacrylate) (PMMA) is attached to graphene grown with CVD on a sacrificial metal substrate, which is then etched away; subsequently, graphene/PMMA is transferred to the target surface, and eventually PMMA is removed by immersion in hot acetone.^28^ Instead of etching away the entire growth substrate, graphene can be delaminated from it by electrochemical bubbling.^29−31^ In addition to these wet transfer methods, there are dry ones, where graphene is again covered with a support layer, e.g., with Poly(vinyl alcohol) (PVA) followed by Poly(dimethyl siloxane) (PDMS) that is used for mechanical delamination of graphene/PVA from the growth substrate.^32^ The PDMS stamp is then detached from the PVA after transfer by heat activation. Finally, the PVA must be removed by immersion in hot water. However, all transfer methods relying on a support layer imply exposure of graphene to chemical solutions.

It is evident that cleaner samples with properties closer to perfect graphene^33−35^ can be achieved through the transfer of graphene in ultrahigh vacuum (UHV). Efforts to exfoliate and transfer graphene in UHV date back to 2012, where highly oriented pyrolytic graphite (HOPG) was pressed onto a Si(111)-(7 × 7) surface, transferring 2 μm × 0.4 μm sized monolayer graphene (MLG) stripes.^36^ However, since HOPG was used as the source, the number of transferred layers was not controlled, and these MLG stripes coexisted with flakes of few-layer graphene (FLG) and of graphite. Large MLG areas could be transferred in high vacuum (10^–6^ mbar) by pressing two MLG/SiC(0001) crystal surfaces onto each other, following a wafer-bonding approach.^37^ Optical microscopy images of both samples after transfer revealed mirror images with MLG (no transfer), bilayer graphene (BLG) (transfer), and no graphene (transfer to the other sample). Due to surface roughness, the transfer yield was only 10% and the transfer took place between identical surfaces. With the aim to synthesize vdW heterostructures of 2D materials under UHV, Sun et al. introduced freshly cleaved 2D materials, such as MoS_2_, WSe_2_, RuCl_3_, black phosphorus, graphene, FeSe, and Bi_2_Sr_2_CuO_6+δ_ into UHV and transferred them onto the target surface by stamping at 300–500 K.^38^ The largest obtained flakes of ML 2D material were 100 μm on a side. Again, as the source wafers were 2D bulk materials, the majority of the transfer was many layers thick. Recently, a multi-UHV chamber system was reported that combines growth and transfer under UHV and offers a mechanical positioning system enabling control over the twist angle and automation of delamination and transfer. Bulk crystals of 2D materials were placed on UHV-compatible Kapton tape and pressed against the SiO_2_/Si(100) target sample.^39^ Also in this study, the largest transferred flakes with uniform thickness were 100 μm a side and several thicknesses coexisted.

Therefore, the existing methods enable either large area ML graphene transfer in ambient conditions or in solutions, or UHV transfer of very small flakes where the thickness is not controlled. Here, we present a method that allows the UHV transfer of large areas (5 × 5 mm^2^) of one monolayer graphene onto target surfaces. As the exfoliation methods described above, it is based on wafer bonding.^40^ However, instead of using 2D bulk materials as the source, we use BLG supported on Poly(tetrafluoro ethylene) (PTFE—Teflon) tape. Teflon is a soft, deformable support that adapts well to the target surface. The weak vdW interaction between the two graphene layers,^41^ compared with the interaction of graphene with Teflon and the target surface, enables the transfer of exclusively the top layer of BLG onto the target surface.

## Methods

### Sample Preparation and Characterization

The preparation of our single-crystal metal surfaces used as target samples for the graphene transfer, as well as Auger electron spectroscopy (AES) and scanning tunneling microscopy (STM) measurements on these surfaces, was carried out in a UHV chamber with a base pressure of *p*_tot_ = 1 × 10^–10^ mbar.^42,43^ Ir(111) and Cu(100) were prepared with repeated cycles of Ar-ion sputtering (*E* = 1.2 kV, *I*_ion_ = 1.2 μA on a spherical sample surface with 7 mm diameter, *t* = 20 min, *T* = 300 K) and subsequent annealing to 1180 °C for Ir and 600 °C for Cu.

Auger electron spectra were acquired between 30 eV and 1000 eV to capture the main peaks of interest, located at 54 eV (Ir), 272 eV (C), and 920 eV (Cu). The primary electron energy, *E*_prim_, the energy increment used for the cylindrical mirror analyzer, Δ*E*_CMA_, and the sample current are given in the figure captions. Prior to each graphene transfer, an AES spectrum was acquired on the target single-crystal surfaces to verify their cleanliness.

Raman spectra were acquired using a Renishaw inVia confocal Raman spectrometer.^44^ We used a laser wavelength of 488 nm with a 2400 lines/mm grating, providing a resolution of 1.3 cm^–1^. Spectra were acquired in two parts, one centered at 1580 cm^–1^ and one centered at 2700 cm^–1^, with an overlap of 200 cm^–1^, used for normalization and the combination of both spectra. The laser power was set to 25 mW with an acquisition time per surface spot of a minimum of 30 s.

For CVD growth of graphene on Ir(111), a clean Ir(111) crystal is heated to 1130–1180 °C in our UHV chamber, raising the base pressure to 5 × 10^–10^ mbar. A partial pressure of 1 × 10^–6^ mbar ethylene (C_2_H_4_) is added for 100 s, the gas is pumped out, and the sample cooled down at a rate of 10 K per second, yielding a single high-quality layer of graphene on Ir(111).^45−47^

For CVD growth of graphene on polycrystalline Cu films, a 20 × 40 mm^2^ sheet is cut from a 25 μm thick 25 × 200 mm^2^ copper foil (Alfa Aesar, purity 99.999%) and cleaned by immersion in acetone for 3 min, followed by Isopropanol for 2 min, and then dried with nitrogen. The Cu sheet is placed in a Carbolite furnace quartz tube, pumped to a pressure of 5 × 10^–3^ mbar. It is then heated to 1031 °C in 4 mbar of H_2_ for 30 min, after which it is exposed to methane (CH_4_) at 18 mbar for 3 min. Subsequently, the methane is pumped out and the Cu sheet is cooled with 10 K/s. Our CVD-grown graphene samples are characterized by Raman *I*(D)/*I*(G) ratios, characterizing the defect density, ranging from 0.10 to 0.25. The graphene quality strongly depends on the growth parameters (temperature and partial gas pressures) and on the roughness, chemical cleanliness, and crystallinity of the Cu sheet. For transfer, we used only the samples showing the best *I*(D)/*I*(G) ratios.

Our Cu etching solution (0.1 M FeCl_3_) was prepared by mixing solid Iron(III) chloride hexahydrate (FeCl_3_·6H_2_O, Sigma-Aldrich) with DI water. For complete etching of the 25 μm thick Cu, the sample was immersed in the solution at room temperature for a minimum of 8 h.

### UHV Transfer Method

**Figure 1:**
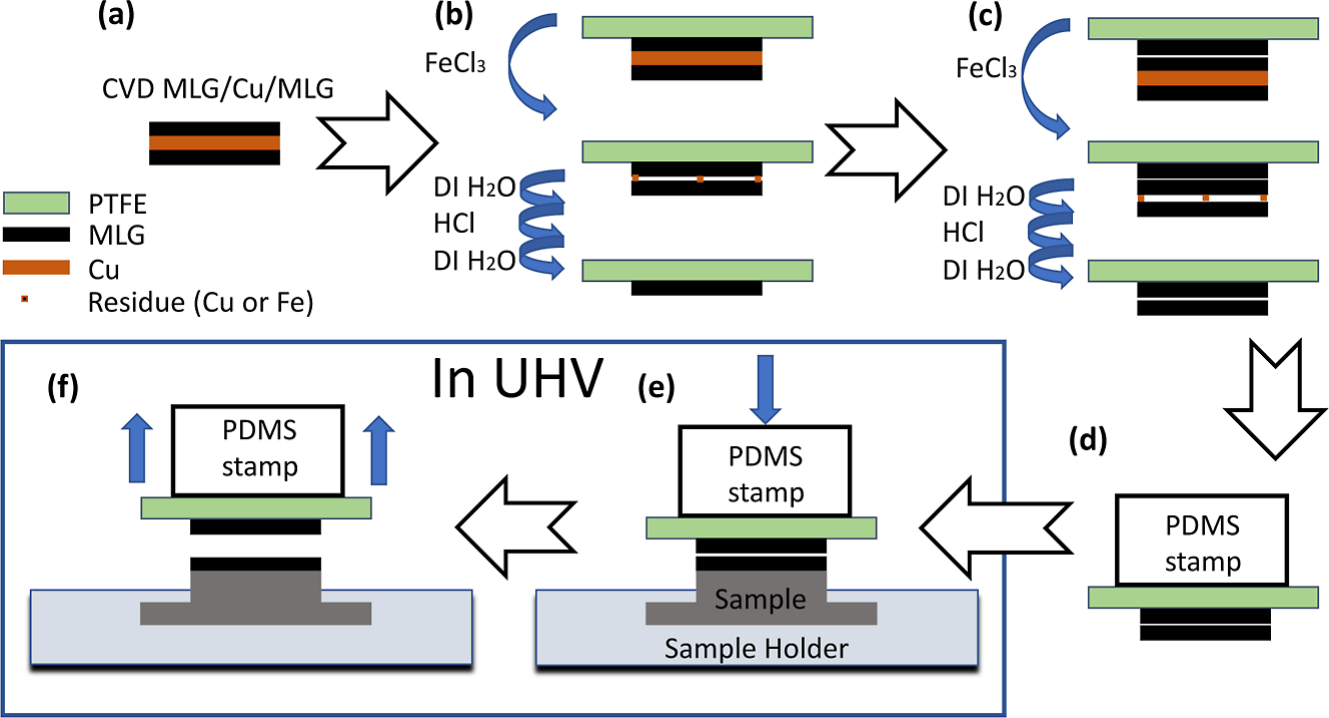
Schematic of our transfer procedure. (a) CVD-grown MLG on both sides of a polycrystalline Cu film. (b) MLG/Cu/MLG supported on the PTFE tape. Etching of Cu in FeCl_3_ solution and rinsing in DI water, followed by etching in HCl and rinsing in DI water removes the bottom layer of graphene and residue leaving a MLG on PTFE. (c) Same procedure as (b), but using MLG/PTFE as a support. Repeating the two etching and rinsing steps leaves a BLG on PTFE. (d) BLG/PTFE attached onto a PDMS stamp and inserted into a UHV chamber. (e) Press on the target sample in UHV. (f) Lift off PDMS/MLG/PTFE leaving behind MLG on the target.

The source ”wafer” of our UHV transfer method consists of high-quality BLG adsorbed onto Teflon tape. In order to prepare it, we grow graphene with CVD on both sides of a 25 μm thick polycrystalline Cu film and cut this film into 5 × 5 mm^2^ large pieces (Figure 1a). We press one such piece by hand for 5 s in ambient environment onto a PTFE tape. The resulting PTFE/MLG/Cu/MLG stack is placed with graphene facing down for 10 h in a 0.1 molar (M) FeCl_3_ solution (Figure 1b). After the completion of this Cu etching, a bilayer of graphene covered by PTFE is left floating on the surface of the solution. This bilayer contains residues of the etching solution between the two layers. Therefore, the stack is rinsed for 1 h in deionized (DI) water. The two graphene layers are weakly bound due to the etching residues and the hydrophobic nature of graphene. Upon contact with the DI water, the change in surface tension causes the bottom graphene layer to break into parts, which then slide apart, leaving behind a single layer of graphene bound to PTFE, as confirmed with Raman spectra taken before and after rinsing. This MLG/PTFE sample is then placed with graphene facing down in a 0.3 M HCl solution for another 10 h to achieve further etching of remaining Cu residue and residual Fe ions from the etching solution. Finally, this sample is rinsed in DI water for 3 h and dried in air. This leaves us with a 5 × 5 mm^2^ MLG square firmly bound to the Teflon tape.

This sample is pressed against a second 5 × 5 mm^2^ large piece of MLG/Cu/MLG (Figure 1c). The two graphene layers are bound by vdW forces. The subsequent Cu etching yields 3 layers of graphene, and again, the layer facing down as well as etching residues are removed in the way described above. Thereby, a high-quality bilayer of graphene on the PTFE tape is obtained. We note that plasma treatment can be used as an alternative method to remove the bottom layer of graphene after Cu etching.^48−50^

The BLG/PTFE stack is subsequently attached with Kapton tape onto a PDMS stamp (Figure 1d) that is then introduced into UHV via a load lock. Using a wobble stick, the BLG/PTFE/PDMS source is aligned parallel to the surface of the single-crystal target sample. The BLG is then pressed against the target surface at 50 °C for approximately 10 s with about 30 N force (Figure 1e). The pressure increase during the transfer process is below 1 × 10^–10^ mbar. Higher temperatures during the graphene transfer led to degassing from the PDMS stamp; we tried temperatures up to 80 °C and found 50 °C to be the optimal compromise increasing binding to the target while minimizing degassing. After lifting off the stamp, practically the entire top layer of graphene is transferred onto the target, while the PTFE with the first graphene layer remains attached to the stamp (Figure 1f). Attempts to transfer directly from a single MLG/PTFE/PDMS did not work, signifying that the MLG adhesion to PTFE is stronger than that to the target.

### AES Graphene Coverage Determination

For samples that have ≤1 ML graphene, we determine the graphene coverage by comparing the AES *I*_C_/*I*_Ir_ intensity ratios to the one of the CVD reference sample. For samples with more than 1 ML of graphene, the AES intensity ratios are no longer proportional to the graphene coverage. For these cases, we use the following AES intensity ratio calculation. We consider the AES sensitivity factors *S*(51) and the mean free paths of Auger electrons emitted from Ir and C, leading to an exponential intensity decay with increasing probing depth. The two relevant mean free paths are the one of electrons emitted by Ir at 54 eV and propagating in graphene, λ_C_(54), as well as the one of electrons emitted from graphene at 272 eV and propagating in it, λ_C_(272). To estimate their values, we use the universal λ(*E*)-curve derived for bulk materials.^52^ The empirical formula 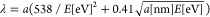 parametrizing this curve uses an effective atomic layer thickness *a*, related to the atomic volume by *V*_atom_ = *a*^3^. We assume the atomic density of diamond for graphene, giving *a*_C_ = 0.207 nm.^53^ This yields λ_C_(54) = 0.322 nm and λ_C_(272) = 0.638 nm.

The AES intensity ratio expected from this for MLG/Ir(111) is^54^
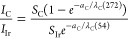
1where *S*_C_ = 0.19.^51^ For Ir, only the AES sensitivity factor of the MNN transition at 1908 eV is tabulated, and we determine the one for the NOO transition at 54 eV^55^ from the published Ir spectrum at *E*_p_ = 3 keV,^51^ yielding *S*_Ir_ = 0.23. For BLG/Ir(111), *a*_C_ is replaced in eq 1 by 2*a*_C_. We obtained *I*_C_/*I*_Ir_(MLG) = 0.435 and *I*_C_/*I*_Ir_(BLG) = 1.42. The first value is far off the experimental value given in Table 1. We attribute this to the different atomic density of graphene and to the approximative character of the empirical formula for the mean free path. However, we trust the relative theoretical value between BLG and MLG on Ir(111) of 3.26 and use it in the Results section to derive the second layer coverage for the BLG transfer sample.

**Table 1:** AES Intensity Ratios of Carbon/Substrate for the Different Growth and Transfer Methods, Graphene Coverage in Monolayers, and Energy Difference between C and the Main Substrate AES Peak

substrate	main peak ratio	coverage [ML]	Δ*E* [eV]
Ir(111) (CVD grown in situ)	1.82 ± 0.06	1.00 ± 0.03	218.0 ± 0.5
Ir(111) (PTFE, pristine)	1.42 ± 0.18	0.78 ± 0.10	220.0 ± 0.5
Ir(111) (PTFE, *T*_ann_ = 1000 °C)	1.62 ± 0.18	0.89 ± 0.10	218.0 ± 0.5
Ir(111) (PVA, *T*_ann_ = 500 °C)	2.26 ± 0.12	1.24 ± 0.07	218.5 ± 1.0
Ir(111) (PVA, *T*_ann_ = 1100 °C)	2.13 ± 0.11	1.17 ± 0.06	217.5 ± 1.0
polycrystal Cu (CVD, *T*_ann_ = 150 °C)	0.57 ± 0.06	1.00 ± 0.10	648.5 ± 0.5
Cu(100) (PTFE, pristine)	0.45 ± 0.05	0.80 ± 0.09	649.3 ± 0.5

### PVA Transfer Method

The transfer method using PVA as the support is not UHV compatible and used for comparison. While this method avoids etching of the Cu growth substrate, since the graphene is delaminated, it requires taking the target sample out of UHV after graphene/PVA transfer in order to remove the PVA from it. For this method, we start by drop-coating the 5 × 5 mm^2^ piece of CVD-grown gr/Cu on the gr side with a single drop of 0.1 M PVA solution at 20 °C. To evaporate the water in the solution, the temperature is slowly (20 °C/min) increased to 70 °C. After 5 min at 70 °C, all water has evaporated and a second drop is deposited on the surface while keeping the temperature constant for another 5 min. This creates a sufficiently thick PVA film that is mechanically stable upon delamination. The sample is then attached with double-sided tape on the Cu side onto an aluminum plate, creating a PVA/gr/Cu/Al stack. The PVA/gr is delaminated from Cu/Al by pressing a PDMS stamp onto the PVA and rapidly lifting the stamp. The gr/PVA/PDMS is then transferred onto the target surface in UHV by pressing. To detach the PDMS, the target sample is heated to approximately 130 °C. For PVA removal, the sample is then taken out of UHV and placed in water at 100 °C for 3 h. The sample is then reinserted back into UHV and annealed before being analyzed with AES and STM.

## Results

We benchmark our UHV transfer method by comparing the quality of graphene transferred onto a target surface with that of CVD grown graphene on that surface. CVD is known to produce extremely well-ordered and clean graphene. Our target surfaces are Ir(111), Cu(100), and polycrystalline Cu. We used AES, STM, Raman spectroscopy, and X-ray absorption near-edge spectroscopy (XANES) to characterize the sample quality. In particular, we determine with the combination of these methods the surface chemical composition, the amount of transferred graphene, the density of structural defects, and the planarity of the graphene.

### Auger Electron Spectroscopy

The quantity of transferred graphene and its chemical cleanliness are characterized by AES. The quantity is inferred from the relative intensities of the main carbon and substrate peaks for samples obtained by transfer compared with reference samples of MLG grown by CVD on both Ir and Cu. The MLG/Ir(111) reference is grown in the same UHV chamber used for transfer, while the graphene on the polycrystalline Cu reference is grown in an external reactor before being inserted into the UHV chamber and annealed to 100 °C to desorb adsorbates.

**Figure 2:**
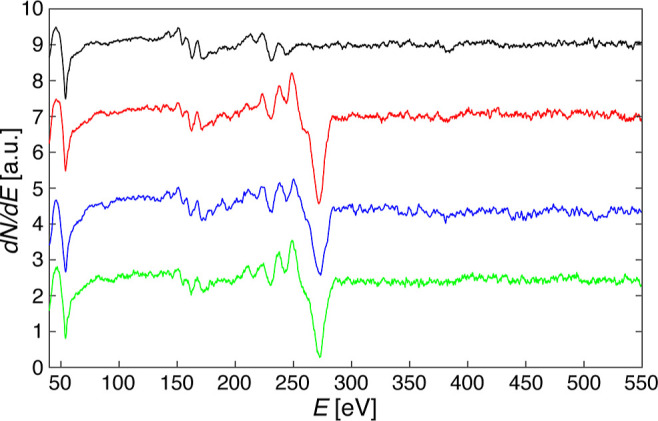
Auger electron spectra obtained on clean Ir(111) (black), on CVD-grown graphene on Ir(111) (red), on gr/Ir(111) after the PTFE-assisted UHV transfer (blue), and after post-transfer annealing to 1000 °C (green). Intensities are normalized to the Ir (54 eV) peak height and spectra are offset for clarity (*E*_prim_ = 3 kV, CMA energy steps Δ*E*_CMA_ = 0.3 eV, *I*_sample_ = 0.65 μA).

Figure 2 shows Auger spectra of clean Ir(111), of the CVD gr/Ir(111) reference sample, and of the MLG/Ir(111) sample obtained by our UHV-transfer method, once immediately after transfer at 50 °C, and once after post-transfer annealing to 1000 °C. Each spectrum is normalized to the intensity of the Iridium main peak located at 54 eV and stemming from NOO Auger transitions.^55^ The background was removed by subtraction of a fourth-degree polynomial.

A first qualitative inspection reveals that the quantity of carbon transferred is close to the CVD reference and that there are no other peaks exceeding the noise, especially no O-related features, the main O_KLL_ Auger peak being at 503 eV^51^ where the spectra are flat. Thus, according to AES, the transfer is clean and the coverage close to a full monolayer (ML). The main C_KLL_ peak is located at 272 eV.^51^ Its shape and energy reveal information about the nature of the C–substrate bond. The C peaks of the sample obtained by transfer postannealing and of the CVD reference are rather similar, especially with regard to their width, the energy of the minimum, and the presence of the shoulder at 260 eV. However, the peak of the pristine sample directly after transfer is broader, its minimum is at slightly higher energy, and the shoulder is almost absent.

Table 1 shows the intensity ratios of the main C to the main substrate peaks, Ir and Cu (920 eV^51^), for transferred and for CVD-grown graphene. The error bars reflect the precision at one surface spot (±0.03 ML) and the spatial variation over a few mm distant surface locations (typically 0 for the CVD-grown samples and up to ± 0.10 ML for samples onto which graphene was transferred). For comparison, we also include the results obtained with our PVA-assisted transfer described in the Methods section above.

We assume a linear relationship between *I*_C_/*I*_Ir_, respectively, *I*_C_/*I*_Cu_ peak intensity ratios, and graphene coverage. C-containing residues will show up as additional graphene coverage. The coverage is given in ML and 1.0 ML is by definition the CVD-grown sample, where a self-limiting full monolayer is formed. On Ir(111), we transfer 0.8 ± 0.1 ML before and 0.9 ± 0.1 ML after annealing. The difference between both values is within the error bars and due to different surface locations being addressed by AES, since the sample is transferred to a different manipulator for the annealing. We exclude annealing-induced segregation of C from Ir bulk since annealing of our clean Ir(111) sample to 1000 °C did not generate AES-detectable amounts of C. Comparison of the peak intensities for the transfer onto Cu(100) to the reference gr/Cu shows that also for this sample we succeed to transfer close to a full graphene ML (0.8 ± 0.1 ML). AES taken at different surface locations reveals that the transfer was successful over the entire 5 × 5 mm^2^ surface area.

The last column of Table 1 shows the energy separation Δ*E* between C and Ir peaks averaged over 5 samples. The error bars reflect the standard deviation between the different samples. Δ*E* = 218.0 ± 0.5 eV for the CVD gr/Ir(111) reference sample, while the pristine gr/Ir(111) samples made with the PTFE transfer method have Δ*E* = 220.0 ± 0.5 eV. Only after annealing to 1000 °C does the sample created by transfer take on the value of the reference sample. The 2 eV chemical shift in the C peak position implies that the pristine transfer and CVD reference sample have different graphene–substrate interactions. Post-transfer annealing is required to obtain the same graphene–Ir(111) bonds that characterize the CVD sample. We elaborate in the discussion section on the likely reasons for this. From Table 1, it is seen that the Δ*E* values for UHV-transferred graphene on Cu(100) and for CVD-grown graphene on polycrystalline Cu agree within the error bars. Thus, transfer onto Cu creates a graphene–substrate interaction similar to that obtained by CVD.

Because the transfer procedure includes the dissolution of the copper support with FeCl_3_ acid, we tested whether any Cu or Fe residues remain on the surface after transfer on Ir(111). High-energy resolution (Δ*E*_CMA_ = 0.1 eV) AES spectra were acquired post-transfer within close ranges of the main Cu (920 eV) and Fe (710 eV) peaks,^51^ but no Cu or Fe signal was detected. The same check has also been performed with the more sensitive XANES technique (see below) on different surface spots with the same result, revealing that both Fe and Cu contaminations are well below 1% ML.

We now compare the performance of our fully UHV-compatible PTFE-assisted transfer method with a PVA-assisted transfer method.^32^ With the latter, we were able to transfer graphene to a target surface both in air (on polycrystalline Cu, SiO_2_, and Al_2_O_3_) and in UHV (on Ni(111) and Ir(111)). However, as stated above, after transfer in UHV, the samples must be exposed to air and placed in hot water for PVA removal, before reinserting it into UHV. The Auger spectrum on the pristine sample revealed very large amounts of carbon and oxygen on the surface, masking almost entirely the Iridium peaks. This large quantity of carbon (along with oxygen) is attributed to residues of PVA which are not entirely removed during the immersion in hot water. Annealing to 500 °C for 15 min partially removes the PVA residues, yielding a *I*_C_/*I*_Ir_ ratio of 2.26, which is still above the reference value of 1.82 (see Table 1). Even upon annealing to 1100 °C, PVA residues are still present, with an excess of about 20% of amorphous C remaining on the surface, as shown by the *I*_C_/*I*_Ir_ ratios and graphene coverage equivalents in Table 1. The O content of the PVA residues is below the AES detection limit for *T*_ann_ ≥ 180 °C.

### Scanning Tunneling Microscopy

**Figure 3:**
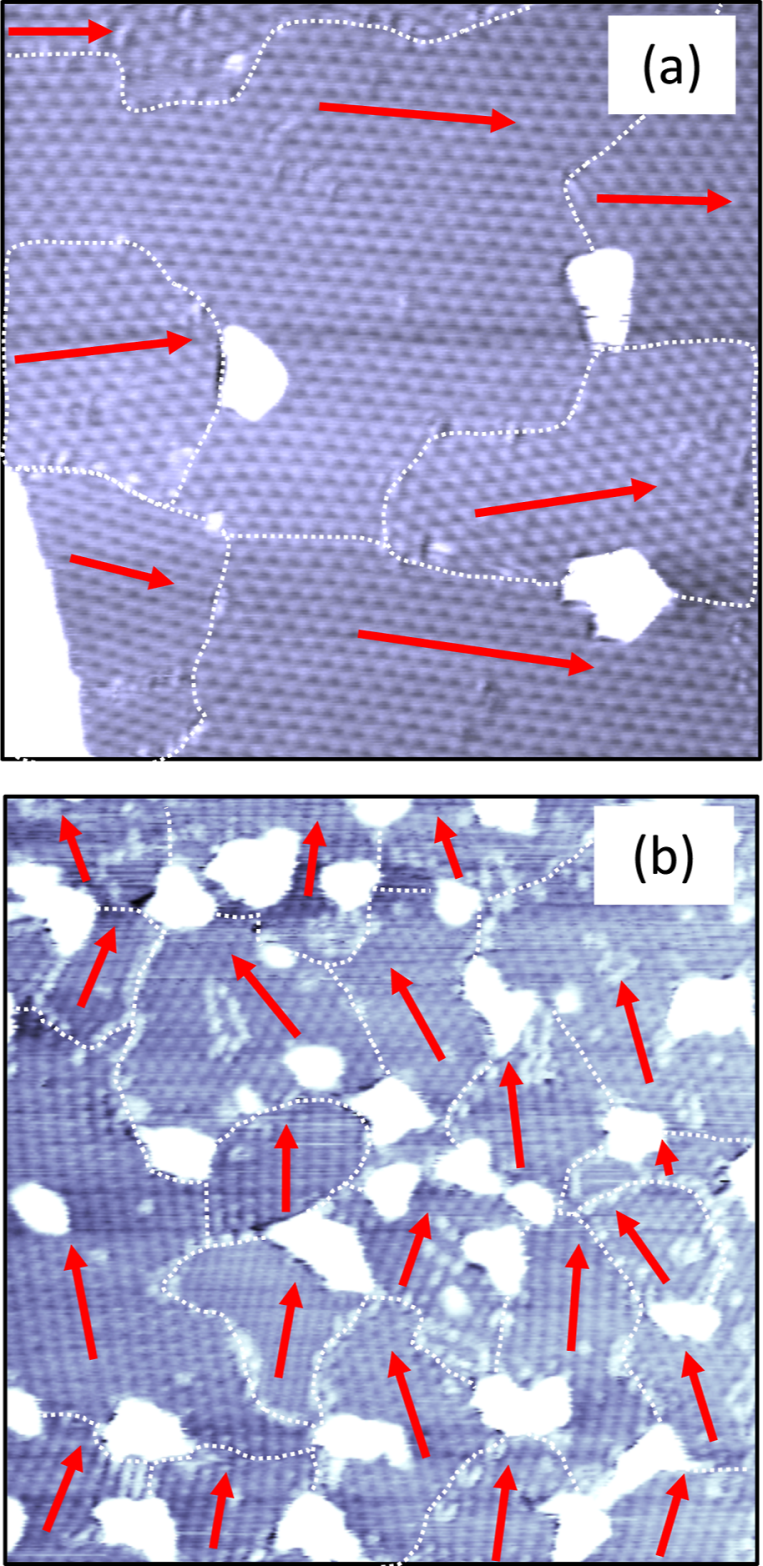
STM images of UHV-transferred graphene on Ir(111). (a) PTFE-assisted transfer method (100 × 100 nm^2^, *V*_t_ = 0.3 V, *I*_t_ = 10 nA). (b) PVA-assisted transfer method (120 × 120 nm^2^, *V*_t_ = – 1.4 V, *I*_t_ = 300 pA). In both cases, *T*_transf_ = 50 °C and *T*_ann_ = 1000 °C. White dotted lines mark moiré domain boundaries and red arrows their orientations.

STM images taken in situ after UHV PTFE-assisted transfer onto Ir(111) and annealing to 1000 °C (see Figure 3a) show that the surface is mainly covered by a moiré pattern with a lattice constant close to the one of 25.2 ± 0.4 Å expected for the (9.32 ± 0.15 × 9.32 ± 0.15) moiré pattern formed by CVD-grown graphene on Ir(111).^46,56,57^ Multiple domains of the moiré pattern can be seen, and their walls are marked with white dotted lines. A finite domain size is expected for CVD-grown graphene on polycrystalline Cu^58^ used as a source for the UHV transfer. The standard deviation of the different domain orientations is 8°. Within the domains, the moiré pattern slightly changes its orientation as it adapts to the orientation of the domain boundaries.

A fraction of the surface (2% in this STM image) is covered by residue appearing as white islands with a mean apparent height of about 10 Å (the white part in the lower left corner is the next higher atomic terrace). The residue mainly agglomerates at domain boundaries. Statistical analysis of the residue density in 30 STM images acquired on different surface areas and on 7 different samples shows an average residue surface coverage of 4%. The O, Cu, and Fe contents of these residues are below the detection limit of our AES and for the samples investigated with XANES also below the detection limit of this method, suggesting that they are mainly composed of amorphous carbon. From apparent height profiles, it is difficult to discern whether the residues are islands above graphene or whether they are buried below graphene, as in the case of Xe inclusions below graphene that were stable against annealing up to 1030 °C.^59^

We were unable to observe the moiré structure for annealing temperatures below 1000 °C. Therefore, the change of the C–substrate interaction detected by AES coincides with the appearance of the moiré pattern in STM. Our interpretation is that the transferred layer is only vdW bound, while annealing creates the moiré pattern exhibiting areas within the unit cell that are covalently bound to Ir.^60^

For comparison, we show in Figure 3b graphene transferred onto Ir(111) with the PVA-assisted method and similarly annealed to 1000 °C after the removal of PVA outside UHV. There are approximately 20 moiré domains characterized by different periods and orientations. The standard deviation of the domain orientations is with 20° significantly larger, and their average size with 700 nm^2^ significantly smaller than that of the sample created with our fully UHV-compatible PTFE-assisted transfer method. In addition, large residue islands with an apparent height of 15 Å are localized at the boundaries of graphene domains and cover roughly 15% of the surface. Thus, the PVA-assisted transfer yields smaller domain sizes, much more residues, and is not UHV compatible.

### X-ray Absorption Spectroscopy

**Figure 4:**
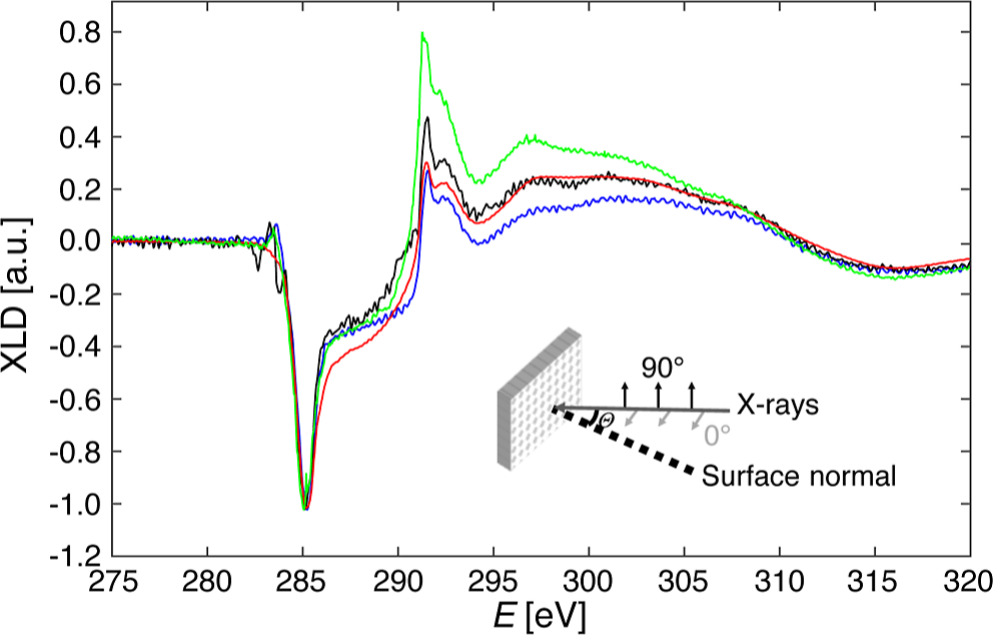
XLD spectra of graphene on Ir(111) grown with CVD (two different samples in black and blue) and created by PTFE-assisted transfer (high quality in red and low quality in green). The π-orbital peak is at 285 eV and the σ-orbital peak at 292 eV. The spectra were acquired at Θ = 60° incidence at room temperature and normalized to the π-intensity.

Carbon atoms in graphene are characterized by in-plane σ orbitals from sp^2^ hybridization and by out-of-plane π orbitals.^61^ XANES allows a selective detection of both orbitals by linearly polarized X-rays and thus to check whether the transferred graphene is flat or wrinkled before the annealing necessary to create the moiré pattern. For the geometry shown in the inset of Figure 4, 0° polarization enhances the sensitivity for σ-orbitals and 90° polarization the one for π-orbitals. Figure 4 shows XANES spectra acquired at the EPFL/PSI X-Treme endstation of the Swiss Light Source^62^ in total electron yield mode, at room temperature, and at the K-edge of C. The π-orbital peak is at 285 eV and the σ-orbital one at 292 eV. The spectra are the difference in absorption between the two polarizations, i.e., the X-ray linear dichroism (XLD). To better highlight the differences between the measured samples, all of the XLD spectra have been normalized to the π-orbital peak intensity.

We prepared one sample where we intentionally wrinkled the PTFE support, which is expected to create undulated graphene and thus referred to as a low-quality (LQ) sample, and compare it with a high-quality (HQ) sample obtained from graphene transfer with a flat PTFE support. These samples were exposed to air on their way from our laboratory to the synchrotron. Therefore, prior to our measurements, they were annealed to 150 °C once inserted into the UHV chamber of the endstation. For comparison, Figure 4 shows two in situ CVD-grown graphene on Ir(111) reference samples. The XLD spectra of the two samples differ slightly, either because different crystals were used or because of the 2–3° uncertainty in the alignment of the X-ray incident angle with the surface normal. Within the errors, the spectrum of the HQ PTFE-assisted gr/Ir(111) is identical with the two CVD reference samples. In contrast, the LQ PTFE-assisted gr/Ir(111) exhibits a significantly more intense σ-orbital peak, indicative of undulated graphene. Therefore, our UHV transfer method yields flat lying graphene, also before annealing.

### Raman Spectroscopy

Defect-free graphene is characterized by two Raman bands, the G band, located at 1580 cm^–1^ and caused by zone-center phonons, and the 2D band involving two *K*-point phonons and located at 2700 cm^–1^. Defects give rise to a D band at 1350 cm^–1^ caused by the phonon scattering from them. Consequently, the intensity ratio *I*(D)/*I*(G) is an indicator of the graphene defect density.^63−67^ We limit ourselves in this subsection to Cu target samples since the fluorescence of Ir samples entirely masks the graphene Raman signal.^7^

**Figure 5:**
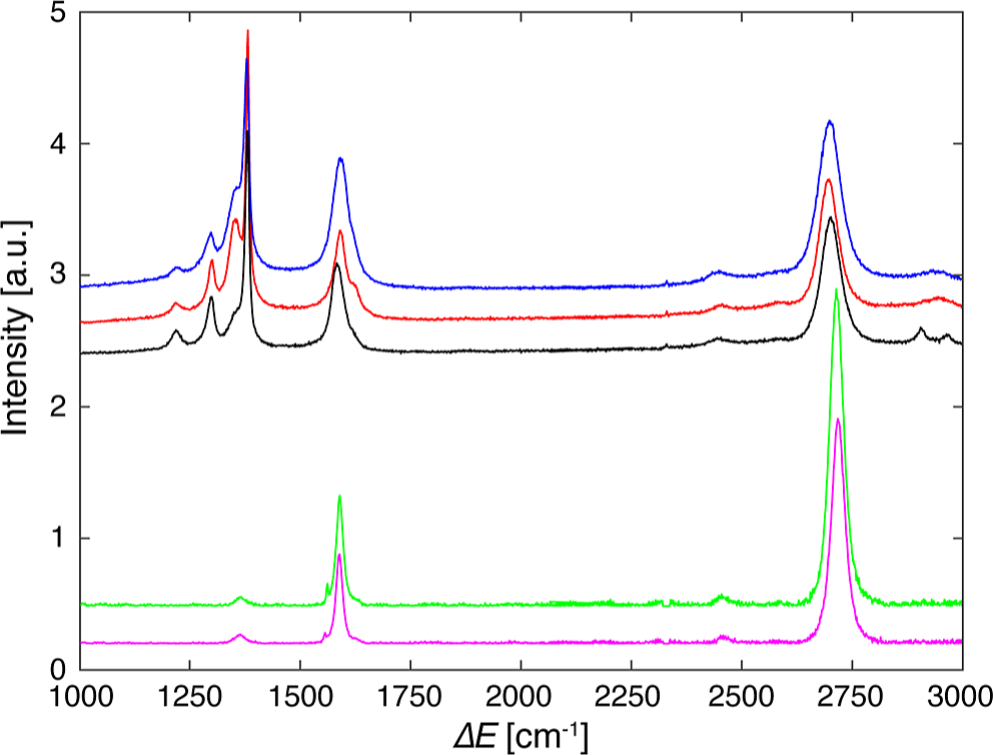
Raman spectra of CVD-grown graphene on Cu (green), PTFE-assisted transferred graphene on Cu(100) (magenta), MLG/PTFE pretransfer (red), BLG/PTFE pretransfer (blue), and MLG/PTFE post-transfer of the top graphene layer in UHV (black) (λ = 488 nm, grating 2400 lines/mm, averaged over 5–8 sample regions).

The two lower spectra in Figure 5 show the comparison between PTFE-assisted UHV transfer of graphene onto Cu(100) (magenta) and CVD-grown graphene on amorphous Cu (green). The two spectra have been normalized to the D band intensity; thus, larger G and 2D peaks signify less defects. The only qualitative difference that can be perceived is that the 2D band has slightly more intensity in the CVD sample.

The three upper spectra in Figure 5 characterize graphene throughout the different transfer steps. They were acquired on the first layer of graphene on PTFE (red), the graphene bilayer on PTFE after the second etching of gr/Cu (blue), and the remaining graphene monolayer on PTFE after transfer of the top layer onto Cu(100) (black). The intense multipeak feature of PTFE, with its largest peak at around 1380 cm^–1^, partly masks the defect peak (D) for these three samples. It shows up as a shoulder to the left of the main PTFE peak. Qualitatively, the MLG/PTFE before transfer has the highest D peak intensity, whereas BLG/PTFE and MLG/PTFE after transfer have a very small D peak. All spectra show a small peak located at 2450 cm^–1^, which is attributed to an overtone between a band around 1100 cm^–1^ and the D band.^68^ All spectra are offset for the sake of clarity.

For a quantitative analysis, the average peak intensity ratios and full width at half-maximum (fwhm) for the spectra shown in Figure 5 are given in Table 2. The peaks were fitted by Lorentzian or Gaussian functions (best fit chosen) after background removal and normalization to the area under the 2000–2200 cm^–1^ region. Error bars are the standard deviations of values measured at different spots of the same sample and therefore give the spatial spread. Due to the large PTFE feature, we refrained from a quantitative analysis of the D peak for the upper three curves.

**Table 2:** Raman Characterization of Graphene during and after UHV Transfer^a^

sample	Δ*E*(2D) [cm^–1^]	Δ*E*(G) [cm^–1^]	*I*(G)/*I*(2D)	*I*(D)/*I*(G)
CVD-grown gr/Cu (step a)	35 ± 3	18 ± 2	0.35 ± 0.05	0.10 ± 0.03
MLG/PTFE (step b)	52 ± 2	37 ± 2	0.72 ± 0.06	
BLG/PTFE (step c)	64 ± 1	47 ± 1	0.79 ± 0.05	
MLG/PTFE post transfer (f)	51 ± 3	30 ± 2	0.71 ± 0.06	
gr/Cu(100) (step f)	39 ± 3	19 ± 2	0.39 ± 0.06	0.12 ± 0.04

a Δ*E* is the fwhm of the respective peak. For the upper three curves, the intense multipeak PTFE feature with its mean peak at around 1380 cm^–1^ makes a quantitative analysis of the *I*(D)/*I*(G) ratio unreliable. The difference in *I*(G)/*I*(2D) ratios for the same number of graphene layers on Cu and on PTFE is an effect of the underlying substrate.^69−71^

The fwhm of the 2D band increases from 52 cm^–1^ for MLG/PTFE to 64 cm^–1^ for BLG/PTFE and decreases to 51 cm^–1^ after the top layer has been transferred. The *I*(G)/*I*(2D) ratios follow the same trend, increasing from 0.72 for MLG/PTFE to 0.79 in BLG/PTFE and decreasing to 0.71 for MLG/PTFE post-transfer. An increase in both the fwhm of the 2D band and the *I*(G)/*I*(2D) ratio has been attributed to an increase in the number of graphene layers.^71,72^ Therefore, these numbers demonstrate that we have MLG/PTFE after the first step and BLG/PTFE after the second step and that the entire top layer of the bilayer graphene attached to the PTFE stamp is transferred to the target surface during the transfer procedure. The G band fwhm follows the same trend, increasing from 37 cm^–1^ for MLG/PTFE to 47 cm^–1^ for BLG/PTFE and reducing back to 30 cm^–1^ post-transfer.

The relative defect peak intensity increases slightly for gr/Cu(100) produced with UHV transfer compared to CVD-grown gr/Cu. However, this increase is within the error bars that reflect the spatial spread. Therefore, the structural quality of UHV-transferred graphene is comparable to that of the CVD grown on polycrystalline Cu. Also, the spatial spread is comparable, showing that our transfer method does not stress the graphene layer inhomogeneously thanks to the flexibility of the PTFE support layer and the PDMS stamp. The *I*(G)/*I*(2D) ratio is identical for both the CVD-grown and -transferred sample, implying that a full monolayer is transferred.

### Creating Bilayer Graphene by Transfer

When two graphene layers are stacked on top of each other with a small twist angle, they form a moiré pattern that gives rise to remarkable electronic and optical properties. Depending on the twist angle, van Hove singularities,^9^ superconductivity,^10^ and flat bands^15^ emerge. BLG can be created by C surface segregation^73^ or by CVD growth with a large exposure of the sample to the precursor gas.^61^ However, to reach the respective twist angles, graphene transfer is indispensable. We demonstrate the creation of BLG by transferring a single layer of graphene with our UHV-transfer method onto CVD-grown MLG/Ir(111).

**Figure 6:**
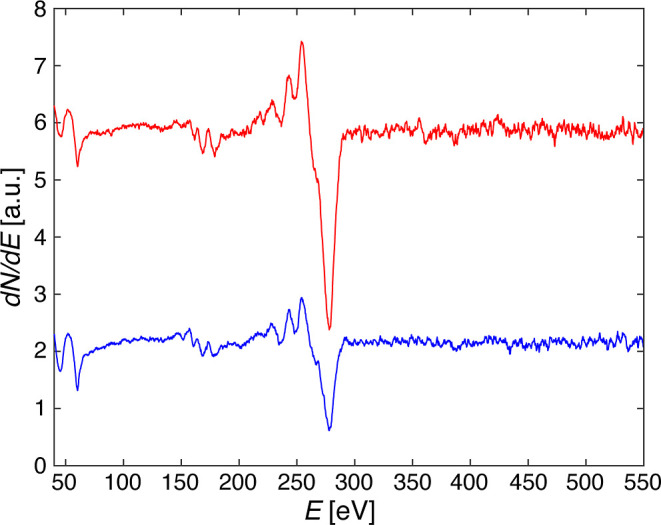
AES spectra of CVD-grown gr/Ir(111) reference (blue) and of BLG/Ir(111) (red) created by PTFE-assisted transfer of graphene onto it. The intensity is normalized to the Ir (54 eV) peak height (*E*_prim_ = 3 kV, Δ*E*_CMA_ = 0.3 eV, *I*_sample_ = 0.65 μA).

The Auger spectrum of the CVD-grown MLG/Ir(111) sample used as a starting substrate is shown in blue in Figure 6. After applying our PTFE-assisted method to transfer graphene onto that sample, we obtain the Auger spectrum shown in red. Its *I*_C_/*I*_Ir_ ratio is 2.2 ± 0.1 times larger, and the sample is again free of oxygen-containing impurities. Considering the escape depths of the respective Auger electrons,^52^ adding a full layer of graphene should increase the *I*_C_/*I*_Ir_ AES ratio by a factor of 3.26 (see Methods section for details of this estimate). Thus, we transferred 53 ± 4% of a graphene monolayer onto the CVD-grown monolayer. The fact that we transfer in this case, where the target surface is already covered by a graphene monolayer, only half of a monolayer is in perfect agreement with the similar binding energy of the graphene layer to the source and target wafer.

**Figure 7:**
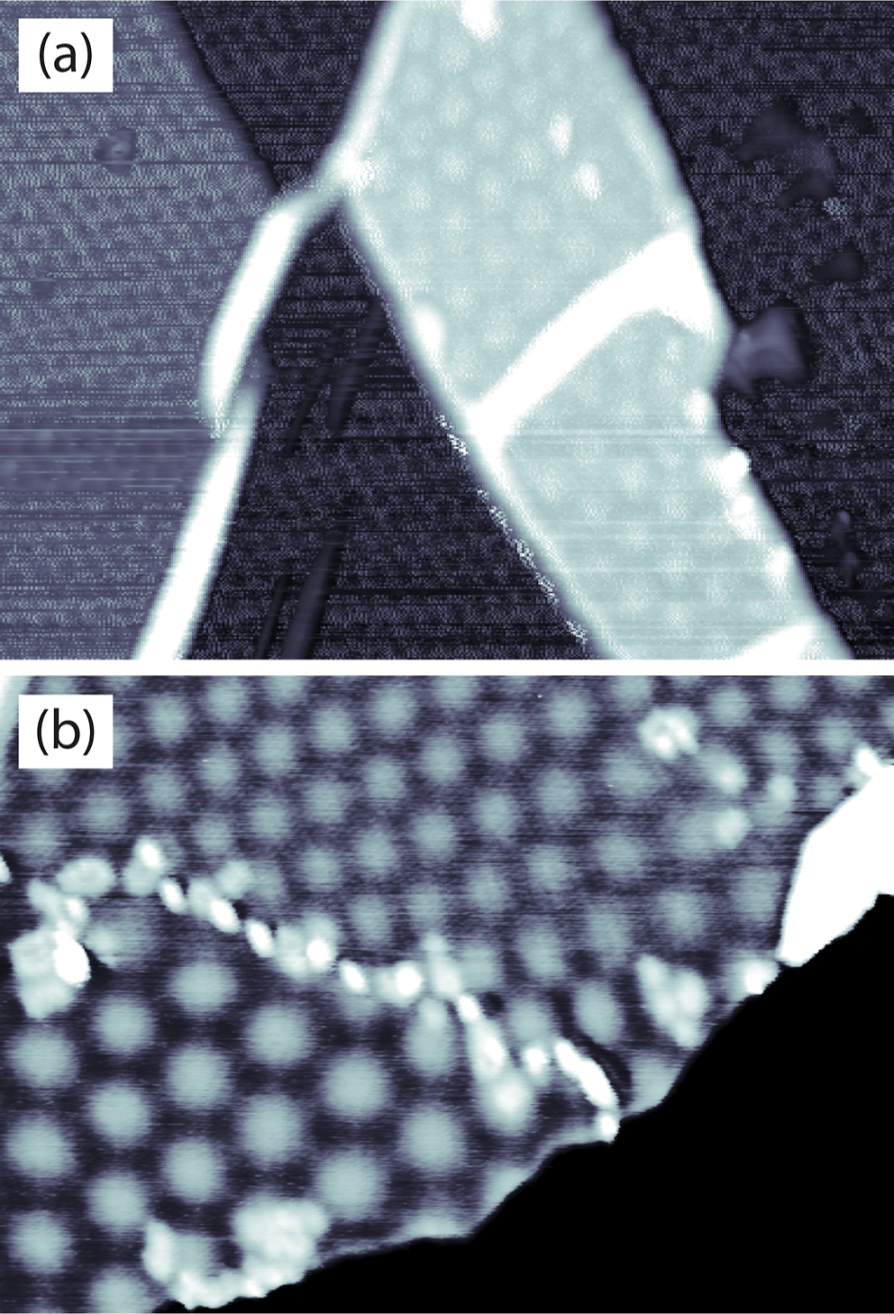
STM images of BLG/Ir(111) on two different sample locations: (a) (88 × 67 nm^2^, *V*_t_ = 1.9 V, *I*_t_ = 1 nA) and (b) (97 × 69 nm^2^, *V*_t_ = 0.7 V, *I*_t_ = 1 nA). For both, *T*_ann_ = 300°. In (b), the color scale has been optimized to reveal the contrast of the BLG moiré patterns. The black part corresponds to MLG.

Figure 7 shows two STM images of this sample. In Figure 7a, the MLG moiré with a period of 25.3 Å covers most of the surface, one brighter terrace on the left and the larger and darker terrace, one Ir(111) step lower lying terrace, on the right. The protruding stripe in the middle of the right terrace has a moiré pattern period of 47.6 Å and is attributed to twisted BLG (tBLG). The apparent height of 5.0 Å with respect to MLG is larger than the geometrical distance, which we attribute to a difference in electronic structure between the two layers.

In Figure 7b, the MLG is on the lower right corner and imaged 5.0 Å below the rest of the surface covered by BLG. The MLG appears black since the color code has been optimized to reveal the contrast of the BLG. The lower left domain of BLG has a moiré period of 99 ± 1 Å, while the upper right has a different twist angle, yielding a moiré period of 68 ± 1 Å. These results are in agreement with bilayer graphene created by high-temperature C deposition onto a CVD-grown graphene layer on Ir(111).^74^ The moiré periodicities we find have been calculated for tBLG on Ni(111).^75^ In both images, there are bright stripes that we attribute to graphene wrinkles and white point defects that we attribute to residues. Their surface coverage is much smaller than that for MLG transfer on Ir(111). A better control of the BLG domain size, as well as of the twist angle between the two graphene layers, can be obtained by starting with a higher quality graphene with respect to the one obtained on polycrystalline Cu.

## Discussion

Our wafer-bonding approach, using graphene bilayers supported on Teflon tape as a source, enables transfer of close to a full monolayer graphene onto single-crystal surfaces under UHV conditions. On Cu substrates, this layer binds as strongly as CVD-grown graphene, while on Ir(111), we have to anneal to 1000 °C to form the moiré pattern obtained by CVD growth. We attribute the need for annealing to a combination of the two effects.

First, while CVD-grown graphene/Ir(111) appears in only four discrete rotational orientations,^76^ transferred graphene arrives with many small domains of arbitrary rotational orientation, reflecting the crystallographic orientations of the grains of the polycrystalline Cu film used for growth. Annealing might be needed to melt these small domains into larger ones, as well as to reorient them such that a moiré structure with strong graphene substrate binding can form. Evidence for this is the fact that the PVA-assisted transfer method gives smaller domains and a larger spread of angles since the large amount of residues located at the domain boundaries prevents melting into larger domains. For graphene islands on Ir(111), the critical size to develop the moiré structure is only very few moiré unit cells,^47,77,78^ from which we conclude that the domain reorientation is more important than their size increase.

The second reason why annealing is needed to form the moiré pattern is that the lattices of graphene and Ir(111) presumably need to be commensurate in order to lock into the moiré structure. Graphene and Ir(111) have very different linear in-plane thermal expansion coefficients, α, creating a temperature-dependent lattice mismatch. For Ir, α_Ir_ is positive and strongly temperature dependent.^79^ Taking its mean value α_Ir_(500 °C) = 7.548 × 10^–6^/K for the temperature range of interest (20 °C–1000 °C), the Ir(111) nearest neighbor distance increases from *d*_nn_ = 271.50 ± 0.02 pm at room temperature^80^ to *d*_nn_ = 273.51 ± 0.02 pm at 1000 °C. Freestanding graphene has a negative linear in-plane thermal expansion coefficient due to transversal acoustic (ZA) phonon modes.^81^ However, this degree of freedom is strongly reduced by binding to the substrate. Consequently, very small but still negative values have been reported for supported graphene, such as α_gr_ = −0.6 ± 0.5 × 10^–6^/K for graphene adsorbed onto octadecyltrichlorosilane-coated glass.^82^ This is more than 10 times smaller than α_Ir_. It is therefore a very good approximation to assume α_gr_ = 0, with which we obtain perfect lattice match at 1000 °C. At this temperature, 9 Ir unit cells lock into 10 unit cells of graphene, as 9/10 *d*_nn_ of Ir at 1000 °C is 246.16 ± 0.02 pm, matching exactly the lattice parameter of graphite, *a* = 246.17 ± 0.02 pm.^83^ After cooling down, the lattice parameter of gr/Ir(111) becomes with *a* = 245.2 ± 0.4 pm slightly smaller, and the unit cell gets slightly larger with 9.32 ± 0.15.^46^ Note that the epitaxial relationship of gr/Ir(111) depends on the CVD growth temperature,^84^ supporting the idea that lattice match under growth, respectively, annealing after UHV transfer at close to room temperature, are important for the resulting structure.

For BLG, annealing to 300 °C suffices to form the various moiré patterns of the tBLG domains. Between two graphene layers, there is no temperature-dependent lattice mismatch, and the annealing is only needed to remove residual adsorbates and to induce a homogeneous vdW bond between the two layers.

## Conclusions

We present a method for transferring macroscopic areas of monolayer graphene in UHV to single-crystal target samples. STM, Auger, Raman, and X-ray absorption spectroscopy demonstrate that the transferred graphene is of comparable quality to CVD-grown graphene. Our approach requires the preparation of the BLG/Teflon tape source wafer outside vacuum. We expect that source wafers of other 2D materials that use similar growth and transfer techniques can be produced. Therefore, our method is expected to work also for transition metal dichalcogenides or *h*-BN.

The distinction from competing UHV transfer methods that are optimized toward device fabrication is that we transfer a single 5 × 5 mm^2^ large graphene monolayer and not small flakes of undefined thickness.

Our approach opens up the creation of stacks of 2D materials in sequences that cannot be achieved by intercalation and with a cleanliness that can only be obtained in UHV. We further anticipate that chemically sensitive samples can be capped with clean 2D materials for their sealing or for the creation of composite materials, employing sequences of epitaxial growth and transfer of 2D materials. One example is growing self-assembled magnetic nanostructures^85,86^ or even superlattices of single-atom magnets^87^ into the third dimension by alternations of transfer and epitaxial growth.
